# The emerging roles of N6-methyladenosine RNA modifications in thyroid cancer

**DOI:** 10.1186/s40001-023-01382-2

**Published:** 2023-11-01

**Authors:** Xiaoxin Xu, Jiayao Zhao, Mingyue Yang, Lutuo Han, Xingxing Yuan, Wencheng Chi, Jiakang Jiang

**Affiliations:** 1 Heilongjiang University of Chinese Medicine, Harbin, 150040 Heilongjiang People’s Republic of China; 2 The Second Affiliated Hospital of Heilongjiang University of Chinese Medicine, Harbin, China; 3 Heilongjiang Academy of Traditional Chinese Medicine Science, No. 33 of West Dazhi Street, Harbin, 150001 Heilongjiang People’s Republic of China; 4 The First Affiliated Hospital of Heilongjiang University of Chinese Medicine, Harbin, China

**Keywords:** Thyroid cancer, N6-Methyladenosine, Progression, Treatment, Prognosis

## Abstract

Thyroid cancer (TC) is the most predominant malignancy of the endocrine system, with steadily growing occurrence and morbidity worldwide. Although diagnostic and therapeutic methods have been rapidly developed in recent years, the underlying molecular mechanisms in the pathogenesis of TC remain enigmatic. The N6-methyladenosine(m6A) RNA modification is designed to impact RNA metabolism and further gene regulation. This process is intricately regulated by a variety of regulators, such as methylases and demethylases. Aberrant m6A regulators expression is related to the occurrence and development of TC and play an important role in drug resistance. This review comprehensively analyzes the effect of m6A methylation on TC progression and the potential clinical value of m6A regulators as prognostic markers and therapeutic targets in this disease.

## Introduction

Thyroid cancer (TC) is the most common endocrine tumor with high morbidity, contributing to a leading cause of death in patients with endocrine malignancy [1]. Presently, thyroidectomy, radioiodine therapy, and thyroid stimulating hormone suppression with l-thyroxin are the main therapeutic schedules for TC, which fulfills a favorable prognosis with a 5 year survival rate of approximately 98% [2, 3]. However, a fraction of patients still experience cancer recurrence, lymph node metastasis, and drug resistance; moreover, a subset of tumors progress to exhibit more aggressive behaviors, which are largely responsible for TC-associated deaths [4]. To monitor the progressive course of this disease, various prognostic factors are tested for clinical application according to age, histological types, and primary tumor sizes [5]. However, these prognostic factors have some limitations owing to their low sensitivity and specificity. Thus, a better comprehension of the molecular mechanisms underlying TC progression is crucial for developing potential biomarkers and identifying novel therapeutic targets for this disease.

N6-Methyladenosine (m6A) is the most abundant epigenetic modification in RNAs, including messenger RNA (mRNA) and noncoding RNA (ncRNA), and affects multiple steps of RNA metabolism, ranging from splicing, export, translation to degradation, thus regulating gene expression [6, 7]. This process is mainly catalyzed by three types of enzymes, also known as m6A regulators, including methylases (writers), demethylases (erasers) and m6A-binding proteins (readers), which make the m6A modification dynamic and reversible [8]. Growing evidence demonstrates that m6A modification is a vital process of cancer biology for regulating carcinogenesis, tumor progression, and drug resistance [9, 10]. Dysfunction of m6A modification has therapeutic potential in cancer management since it can be exploited as a prognostic biomarker or a therapeutic target [11, 12]. Therefore, the study of m6A modification has a great significance in pathogenesis, prognosis evaluation, and therapeutic options of TC.

Recent studies have shown that m6A modification is dysregulated in TC, which plays crucial roles in tumor occurrence and progression [13–15]. This review briefly summarizes the modulation of m6A modification of RNAs, and emphasizes the effects of dysregulated m6A modification and abnormally expressed m6A regulators in TC progression and discusses the potential of m6A modification as prognostic tools and therapeutic targets for this disease.

### The regulation of the m6A modification of RNA

m6A modification is an evolutionarily conserved epigenetic regulation process and is widely distributed among mRNAs and ncRNAs, including micro (mi) RNAs, long noncoding (lnc) RNAs and circular (circ) RNAs [16–18]. RNA m6A modification refers to a methyl group is attached to the N6 position of adenosine and tends to occur in DRACH (*D* = G/A/U, *R* = G/A, and *H* = A/C/U) [19]. The most common motif is GGACU, which is in the 3′-untranslated regions (UTRs), coding sequences, around stop codon regions, and 5′-UTRs [20]. It is a reversible process and maintains a state of dynamic equilibrium that is mainly mediated by highly conserved regulatory proteins, including methylases (writers), demethylases (erasers) and m6A-binding proteins (readers) [21].

### m6A writers

The m6A methylation is mainly catalyzed by "writers" methyltransferase complexes, comprising methyltransferase‐like 3 (METTL3) and methyltransferase‐like 14 (METTL14), which combine with each other to form a stable METTL3–14 complex [22]. As the core subunit of this complex, METTL3 can bind to the methyl donor S-adenosylmethionine (SAM), while METTL14 serves as an allosteric activator of the METTL3 and mediates the recognition of RNA substrates by METTL3 [23]. Wilms' tumor 1-associating protein (WTAP) is the cofactor of this complex to promote its localization to nuclear speckles enriched with various RNA processing factors [24]. The vir‐like m6A methyltransferase associated (KIAA1429, also known as VIRMA) is regarded as another isoform of methyltransferase complex and recruits catalytic core components (METTL3/METTL14/WTAP) to guide regioselective m6A methylation [25]. Similarly, RNA‐binding motif protein 15 (RBM15) and its paralog RBM15B, bind and recruit “writer” complexes to specific sites [26]. Besides, as a crucial component of this complex, zinc finger CCCH domain‐containing protein 13 (ZC3H13) can anchor the methyltransferase complex in the nucleus [27]. As series of homologs of METTL3, METTL4 has been identified as m6Am (2’O-ribose methylated m6A) methyltransferase of predominantly small nuclear RNAs [28], METTL5 is a novel methyltransferase responsible for the m6A modification on 18S rRNA [29], and METTL16 controls cellular SAM levels and modifies U6 small nuclear RNA with the m6A mark [30].

### m6A erasers

Demethylation, which is the elimination of the methyl group of the sixth nitrogen atom of adenine on the RNA, is performed by another enzyme family called demethylases (erasers), mainly including obesity-associated protein (FTO) and AlkB homologue 5 (ALKBH5), thus making m6A modification dynamic and reversible [9, 10]. Both FTO and ALKBH5 belong to the AlkB dioxygenase family and rely on the cofactors Fe^2+^ and α-ketoglutarate to catalyze the oxidative demethylation process [31]. FTO initiates the demethylation of m6A by a two-step process in the cytoplasm and nucleus, which generates two intermediates, including N6‐hydroxymethyladenosine (hm6A) and N6‐formyladenosine (f6A). The addition of a hydroxyl group to the methyl group of m6A directly forms hm6A, which can be further oxidized by FTO to f6A. Both hm6A and f6A can be hydrolyzed into adenosine [32]. Unlike FTO, ALKBH5 can directly oxidize m6A to adenosine without an intermediate in the nucleus, indicating its function as an m6A-specific demethylase [33]. Besides, ALKBH3 is a novel m6A demethylase that mainly functions on transfer RNA and improves protein translation efficiency [34, 35]. More m6A demethylases are predicted to exist, and their molecular mechanisms and biological functions remain to be investigated.

### m6A readers

The gene regulatory effects of m6A are mediated by a group of RNA‐binding proteins, also known as readers, which can recognize m6A modification, executing the functions of regulating RNA translation, post-translational modification, splicing, output and degradation [36]. Various m6A reader proteins have been identified in mammals, including YTH domain‐containing family protein 1–3 (YTHDF1-3), YTH domain‐containing protein 1–2(YTHDC1-2), insulin-like growth factor 2 mRNA-binding proteins 1–3 (IGF2BP1-3), heterogeneous nuclear ribonucleoproteins (hnRNPs), and eukaryotic initiation factor 3 (eIF3) [37, 38]. YTHDF1 is verified to recognize and bind to m6A sites of target genes, and subsequently improves RNA translation and protein synthesis efficiency by recruiting translation initiation factors, such as eIF3 [39]. As the first characteristic m6A reader, YTHDF 2 interacts with m6A-modified RNAs to mediate RNA degradation and interrupt the stability of the RNA [40]. YTHDF3 plays a regulatory role in the translation or decay of m6A-modified RNAs in coordination with the activities of YTHDF1 or YTHDF2 [41]. In addition, YTHDC1, which is located in the nucleus, can promote RNA splicing in the nuclear speckle and regulate the export of m6A methylated RNA from the nucleus to accelerate protein expression [42]. YTHDC2 improves the translation efficiency of the substrate by targeting the conserved m6A motif [43]. IGF2BPs strengthen RNA stability, protect m6A-modified RNAs from degradation and promote translation [44]. The HNRNP family of readers contains HNRNPC, HNRNPG and HNRNPA2B1, which bind to m6A sites of RNAs with high affinity. HNRNPC and HNRNPG affect the localization and splicing of RNAs in the nucleus [45], while HNRNPA2B1 regulates m6A by activating the downstream pathway of miRNA primers and the processing of miRNA precursors [46].

**Fig. 1:**
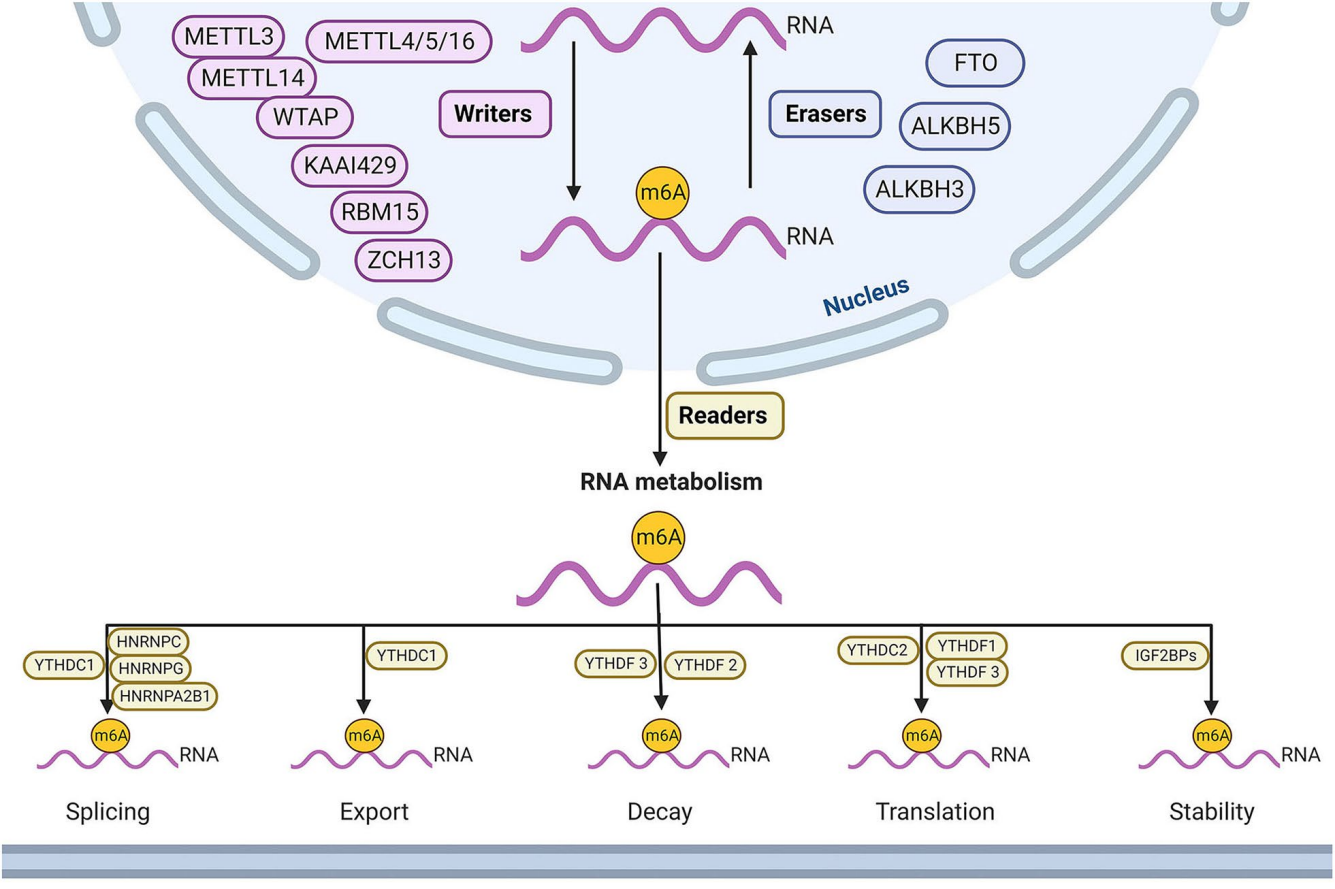
Mechanism of the m6A modification. The m6A-RNA methylation is modulated by writers, erasers and readers, writers mainly contain METTL3–METTL14 complex, WTAP, KAAI429, RBM15, ZCH13 and METTL4/5/16, which promote m6A methylation; erasers include FTO, ALKBH5 and ALKBH, and induce m6A demethylation; readers consist of YTHDF1-3, YTHDC1-2, IGF2BP1-3 and HNRNPs (HNRNPC, HNRNPG and HNRNPA2B1), which are involved in RNA splicing, export, decay, translation and stability

Since its discovery, m6A modification plays a crucial role in physiological and pathological processes including cell differentiation, apoptosis, tumorigenesis, and cancer progression by regulating RNA metabolism (Fig. 1). The m6A modification alters genetic information at the transcriptional and translational level, regulates the expression of RNAs and affects mRNA translation to modulate the expression of target genes. Abnormal expression of m6A regulatory factors changes the m6A methylation level of various genes, which are involved in proliferation, metastasis, and invasion of cancer cells. So, identifying the intricate interaction between writers and erasers, and molecular mechanism of readers on regulating downstream cascades and metabolic processes of RNA, may provide strategies for the treatment of TC. Besides, apart from the aforementioned m6A regulatory factors, other subunits of the methyltransferase complex likely await discovery.

### Effects of m6A in TC progression

The m6A modification and m6A regulatory factors have been increasingly investigated in TC. Generally, some regulators can be employed as tumor suppressors to inhibit the occurrence and progression of TC, while others function as oncogenes (Table 1). Compared with adjacent normal tissues, the abnormal expression of m6A methylation modification regulators in TC has attracted the attention of researchers, since it has been predicted to be of great value in TC tumorigenesis, malignant progression, and metastasis.

**Table 1:** Roles of m^6^A regulators in thyroid cancer

Regulators	Expressions	Targets	Roles	Functions	Mechanisms	References
METTL3	Up	miR-222-3p	Oncogene	Promotes tumor growth and metastasis	Promotes the expression of miR-222-3p and thus downregulates STK4	[48]
METTL3	Up	TCF1	Oncogene	Promotes proliferation and migration	Upregulates the IGF2BP2-mediated m^6^A modification of TCF1mRNA	[51]
METTL3	Down	c-Rel, RelA	Suppressor	Inhibits tumor growth	Inactivates the NF-κB pathway by cooperating with YTHDF2 and further reduces the accumulation of tumor-associated neutrophils	[52]
METTL3	Down	STEAP2	Suppressor	Inhibits PTC proliferation, EMT, migration and metastasis	Enhances the stability of STEAP2 mRNA in an m^6^A-dependent manner and inhibits the Hedgehog signaling pathway	[53]
FTO	Down	TP53	Suppressor	Inhibits tumor progression	Targets m6A methylation of TP53	[60]
FTO	Down	APOE	Suppressor	Inhibits PTC glycolysis and growth	Suppresses the expression of APOE through IGF2BP2-mediated m^6^A modification	[62]
FTO	Down	SLC7A11	Suppressor	Promotes tumor ferroptosis	Downregulates the expression of SLC7A11	[61]
ALKBH5	Down	TIAM1	Suppressor	Inhibits proliferation and promotes ferroptosis	Suppresses the expression of TIAM1 by m^6^A modification	[69]
IGF2BP2	Up	lncRNA HAGLR	Oncogene	Promotes proliferation, migration and invasion	Promotes the expression of lncRNA HAGLR in an m^6^A-dependent manner	[75]

### METTL3 and TC

As the key catalytic subunit mediating m6A modification, METTL3 has been reported to be expressed aberrantly in various tumors, including hematopoietic malignancies and solid tumors, and plays vital roles in tumor progression as an m6A regulator, either as a tumor suppressor or an oncogene [47]. The association between METTL3 and TC has recently been identified. It has been shown that METTL3 silencing potently reduces TC cell viability and proliferation through decreasing total m6A levels in mRNAs, indicating an oncogenic role of METTL3 in TC [13]. In line with this result, Lin et al. reported that METTL3 is significantly elevated in TC tissues and cells and is correlated with poor prognosis of TC patients. METTL3 promotes the proliferation, migration, and invasion of TC in vitro as well as tumor growth and lung metastasis in vivo by augmenting miR-222-3p expression and thereby diminishing the expression of serine/threonine stress kinase 4 (STK4) [48]. Reduced STK4 expression and resultant YAP hypo-phosphorylation promote TC cell proliferation and tumorigenicity via the inactivation of Hippo signaling pathway [49, 50]. Similarly, METTL3 is also highly expressed and can enhance T cell factor (TCF1) protein expression by upregulating the m6A level of TCF1mRNA and recruiting the m6A “reader” protein IGF2BP2. The increased expression of TCF1 leads to abnormal activation of the Wnt signaling pathway, thus promoting proliferation and migration in TC cells [51]. These findings suggest that METTL3 exerts an oncogenic effect in TC, leading to proliferation, invasion, and metastasis. However, sometimes METTL3 also acts as a tumor suppressor in TC. He et al. revealed that decreased expression of METTL3 indicates an unfavorable prognosis in individuals with papillary TC, and METTL3 deficiency accelerates cancer cell proliferation and metastasis. Mechanistically, METTL3 impedes tumor progression through m6A modification on NF-κB mRNA in a YTHDF2-dependent manner, which in turn attenuates IL-8 production of papillary TC cells to recruit neutrophils, ultimately suppressing tumor progression [52]. A similar study reported that upregulating METTL3 improved m6A enrichment on STEAP2 mRNA and promoted its translation, blocking the activation of Hedgehog signaling pathway and epithelial–mesenchymal transition [53]. Thus, METTL3 affects the development of TC through m6A modification. It is reasonable for targeting of METTL3 in the treatment of TC.

In sum, METTL3 is expressed differently in TC and exert dual effects, oncogene or tumor suppressor. It is generally acknowledged that METTL3 exhibits oncogenic functions in most cancer types, such as acute myeloid leukemia, breast cancer, and lung cancer [54]. However, it is also reported that METTL3 serves as a tumor suppressor in TC. How could METTL3 plays paradoxical roles in the same cancer type needs to be carefully investigated and addressed. It should be noticed that METTL3 is found to play the tumor-suppressive function in papillary TC [52, 53]. One explanation may be that experimental outcomes are affected by tumor heterogeneity and different model systems exploited in the study. Thus, future investigations should take histology subtype of TC into consideration for further comprehensive and detailed studies. Besides, several small molecular inhibitors against METTL3 are regarded as promising strategies for cancer persistence and recurrence, and the pharmacological combination of METTL3 inhibitors and chemotherapeutic agents may provide basic principles for future research [55]. However, targeting of METTL3 for clinical application is still in its infancy. Further clarification of the regulation, functions, and mechanisms of METTL3 in TC is urgent.

### FTO and TC

Identified as the first RNA m6A demethylase, FTO has been reported to play critical roles in numerous types of cancers [56]. Abnormality in FTO m6A demethylase activity has been implicated in tumorigenesis, progression, metastasis, and drug resistance [57]. In most cancer types, FTO is upregulated and functions as an oncogene, whereas it also exhibits a tumor-suppressive in certain types of tumors, indicating that FTO function in various cancers is context-dependent [58]. Increasing evidence has demonstrated that FTO is strongly associated with TC progression. The relationship between m6A modification regulators and prognostic value, as obtained from the TCGA database, reveals that FTO is remarkably downregulated in TC specimens and higher FTO levels shows longer overall survival and predicts a favorable prognosis in patients with TC, suggesting a tumor-suppressive role of FTO [14, 59]. Furthermore, Tian et al. observed a significant decrease of FTO expression in the blood and tumor tissues of TC patients, and documented that FTO expression was related to lymph node metastasis and tumor grade [60]. FTO can inhibit tumor progression in TC by mediating ferroptosis, a form of programmed cell death. Researchers confirmed that upregulation of FTO-mediated m6A demethylation suppresses the expression of SLC7A11, an antioxidant acting as negative regulator of ferroptosis, thus facilitating ferroptosis and suppressing the proliferation, invasiveness, and growth of papillary TC in vivo and in vitro [61]. In addition, FTO can also inhibit APOE mRNA stability through m6A modification mediated by IGF2BP2. The reduced expression of APOE leads to abnormal activation of the IL 6/JAK2/STAT3 signaling pathway, thus blocking the glycolytic metabolism and tumor growth in papillary TC [62].

In conclusion, FTO is downregulated in TC compared with the adjacent nontumor tissues and inhibits tumor progression. As mentioned above, FTO is involved in regulating the ferroptosis and glucose metabolism in TC. In addition to these biological processes, FTO also affect tumorigenesis and progression by regulating other hallmarks of cancer, including cancer stem cell self-renewal, tumor microenvironment, and immunity [63]. Thus, further investigations are warranted to fully uncover the functions and underlying molecular mechanisms of FTO in other biological processes of TC. Although the downstream targets of FTO have been identified to some degree, inducive factors of FTO deficiency in TC remain elusive. Previous study unveiled that miRNAs and DNA methylation proteins can serve as upstream factors to govern FTO expression in various tumors [64]. Whether FTO also regulates other downstream genes linked to TC malignancies requires experiments for verification. Given the tumor-suppressive role of FTO in TC, combination of FTO activators with existing cancer therapies, such as chemotherapy, radiotherapy and immunotherapy, holds promising clinical application significance in TC patients.

### ALKBH5 and TC

As the second m6A demethylase, ALKBH5 inhibits a critical function in maintaining a dynamic equilibrium between methylation and demethylation of RNAs. Compared with FTO, ALKBH5 possess higher specificity and affinity in binding to RNAs, as it is equipped with alanine‐rich sequence and coiled‐coil structure in its N‐terminus, which may contribute to its accurate localization and functioning [33, 65]. It is well known that aberrant expression of ALKBH5 both promote and suppress carcinogenesis based on cancer types, via post-transcriptional modulation of oncogenes or tumor suppressors in an m6A-dependent manner, resulting in alterations in cancer cell proliferation, migration, invasion, metastasis, drug resistance, and cancer immunity [66]. In TC, reduced ALKBH5 gene expression is associated with shorter overall survival times, indicating that ALKBH5 could suppress tumor progression [59, 64]. In a most recent study performed by Ji et al., downregulation of ALKBH5 promotes papillary TC mitochondrial oxidative phosphorylation and inhibits glycolysis, and thus participating in cell proliferation, tumorigenesis and tumor growth. Compared to adjacent noncancerous tissues, TC tissues expressed lower levels of ALKBH5. ALKBH5 knockdown promotes glucose uptake in TC xenografts through activation of circNRIP1-mediated glycolytic functions of tumor cells by sponging oncogenic miR-541-5p and miR-3064-5p as well as by upregulating pyruvate kinase M2 (PKM2) levels [67]. Overexpression of PKM2 increases the rate of glycolysis, and subsequently glucose is converted to lactate and ATP is massively produced to flourish TC development [68]. Another deep impressed study unveiled that upregulation of ALKBH5 inhibited cell proliferation and tumor growth in TC. Further mechanistic investigations explained that ALKBH5 regulates the Nrf2/HO-1 axis by mediating m6A modification on TIAM1 mRNA and thus eliciting ferroptosis in TC [69]. These findings imply that ALKBH5 exerts a tumor-suppressive role in TC via glucose metabolism and ferroptosis through m6A modification.

In conclusion, ALKBH5 has a low expression in TC and exhibits a tumor-suppressive effect through m6A modification-mediated epigenetic regulation. Just like the role of FTO in the pathogenesis of TC, ALKBH5 also regulates ferroptosis and glycolysis, thus affecting the tumor progression. Indeed, accumulating evidence confirmed the biological significance of ALKBH5 in tumor immune responses, cancer cell immune evasion and tumor microenvironment remodeling [70]. However, few studies have reported on the regulation of immunity by ALKBH5 in TC. Thus, future studies are certainly required to determine the regulatory mechanisms of ALKBH5 in TC-related immune responses. It is worth noting that that hypoxia, epigenetic modulators, transcription factors, and ncRNAs act as main contributors to ALKBH5 dysregulation in cancer [66]. Thus, upstream regulators mediating downregulation of ALKBH5 in TC remain to be investigated. Since ALKBH5 can halt the TC progression, approaches to upregulate ALKBH5 could represent viable therapeutic strategies for TC. Promising treatment options such as small-molecule modulators, compounds targeting the regulators of ALKBH5, and gene therapy have been expected to manipulate ALKBH5-mediated m6A demethylation in cancer [71]. The application of these therapeutics to restore ALKBH5 expression in TC as well as its clinical efficacy and biosafety await further validation.

### IGF2BP2 and TC

Importantly, m6A modification also affects cancer biology by recruiting the reader protein IGF2BP2, a member of a highly conserved RBP family that regulates a spectrum of processes in RNA metabolism, including localization, splicing, translation, stability, and decay in an m6A-dependent manner under normal and stress conditions [72]. A body of data disclose the causal role of IGF2BP2 in cancer etiology, and find that IGF2BP2 is overexpressed during carcinogenesis and early development, which causes the tumorigenic phenotype, tumor progression and unfavorable prognosis [73]. The expression of IGF2BP2 is found to be higher in tumor tissues than in normal tissues, and it is strongly correlated with disease-free survival and clinical phenotypes in patients with papillary TC [74]. Dong et al. investigated the oncogenic function of IGF2BP2 on TC. In their study, a remarkable IGF2BP2 high expression was observed in TC tumor tissues in comparison with their adjacent noncancerous samples. IGF2BP2 knockdown suppressed TC cell proliferation, cell cycle progression, cell migration and invasion, and induced TC cell apoptosis through downregulating lncRNA HAGLR [75], suggesting IGF2BP2 can directly regulate lncRNAs in an m6A-dependent way. Besides, IGF2BP2 are also required for m6A demethylases-modified m6A methylation in TC. For instance, FTO inhibits cell growth and glycolysis by reducing the APOE mRNA stability and expression through m6A modification mediated by IGF2BP2 [62]. This result indicates that the crosstalk between m6A erasers and readers can affect TC progression.

Altogether, IGF2BP2 facilitates the occurrence and progression of TC through m6A modification. It is established that m6A readers are not only required for m6A writers-catalyzed m6A methylation by forming polymeric methyltransferase complex, but also required for m6A demethylases-modified m6A methylation in cancer biology [76]. Considering IGF2BP2 and FTO combine together to regulate m6A methylation process in TC, further clarifying the molecular mechanism of IGF2BP2 in crosstalk among other writers, readers, and erasers of m6A is important for understanding the pathogenesis of TC. In addition to IGF2BP2-mediated directly regulation on the target mRNA expression, IGF2BP2 also interact with ncRNAs to stabilize or increase mRNA levels [77]. Given the prooncogenic role of IGF2BP2 in TC, its pharmaceutical inhibition could have a crucial therapeutic potential. However, IGF2BP2 and other m6A readers can compete for the same target mRNA, and suppression of IGF2BP2 may contribute to feedback activation of other readers, possibly developing drug resistance [78]. Thus, further investigations are required to verify the effect of IGF2BP2-targeting drugs on TC phenotypes, and to assess the selectivity and efficacy of these agents in the clinical setting.

### RNA m6A as biomarker in TC

Intensive evidence has uncovered that dysregulation of m6A regulators, including writers, erasers, and readers, can act as prognostic markers in TC. These regulators are involved in the tumor progression and function as useful tools to evaluate the prognosis of patients with differentiated TC [79]. Particularly, the expression of HNRNPC is significantly elevated in TC, while WTAP, RBM15, YTHDC2, YTHDC1, FTO, METTL14, METTL3, ALKBH5, KIAA1429, YTHDF1, and ZC3H13 are remarkably decreased in tumor tissues; moreover, among these downregulated regulators, RBM15, KIAA1429, and FTO are used as a reference for prognostic analysis, which shows better performance in predicting the prognosis of TC with high accuracy [59]. This established m6A-related three-gene prognostic model is also independent prognostic markers of overall survival in TC, which modulate the key signaling pathways of TC progression, such as proteolysis and immune response [14, 80]. It is also found that IGF2BP2, STT3A, MTHFD1, and GSTM4 could be a prognostic signature of TC patients to predict disease-free survival [74]. Li et al. further analyzed m6A regulators using The Cancer Genome Atlas databases and reported that the expression of two genes (WTAP and METTL16) are closely related to the histologic grading and TNM stage, and serve as a prognostic indicator for overall survival in TC patients [81]. Of importance, dysregulated m6A regulators can be used in combination as the m6A score model for the prognostic analysis and treatment response, which reveals that patients with lower m6A score have prolonged overall survival and predicts the efficacy of immunotherapy [82]. It has also been shown that m6A regulators can affect the prognostic analysis of TC by modulating specific ncRNAs. For example, the established m6A-related lncRNAs prognostic model acts as a novel predictor and shows good performance in predicting the progression-free survival and recurrence in TC patients [83]. Bioinformation analysis proposed that m6A-related lncRNAs and mRNAs can influence gene mutation, immune cell infiltration and tumor microenvironment in TC [84].

In conclusion, m6A regulators are dysregulated in TC and could be used as independent prognostic factors to optimize patient monitoring. However, there are still challenges in clarifying the optimal candidates for the early diagnosis and screening of TC. In consideration of the limitation of single m6A regulator acting as biomarker in different tumor stages and types, comprehensive analysis should be performed to identify the m6A regulator signatures as diagnostic biomarkers and prognostic indicators for patients with TC. In addition, multiple studies have screened m6A regulators based on analysis of public sequencing databases, reckoning without tumor heterogeneity and pathological types, which leads to drawing biased conclusions. Thus, more attention should be given to large-scale investigations for verifying the specificity and sensitivity of m6A regulators as biomarkers in TC patients.

### RNA m6A as therapeutic targets in TC

In terms of the pivotal role of m6A modification levels and m6A regulators in TC, targeting aberrant expressed m6A regulators may be a potential therapeutic target for this disease. As mentioned above, both FTO and ALKBH5 can impede TC progression, thus restoration or augmentation of their expression levels may provide a potential therapeutic strategy for TC; whereas, IGF2BP2 plays an oncogenic role in TC, so pharmacological inhibition of IGF2BP2 may exert antitumor effects in this disease. However, METTL3 can act either as an oncogene or a tumor suppressor in TC, depending on the specific cellular context, which suggests that approaches to both induce and suppress METTL3 could represent potential treatment options for patients with TC. Furthermore, the m6A modification also plays an essential role in mediating TC cells response to chemotherapy and radiotherapy. Tyrosine kinase inhibitors and radioiodine are the crucial agents for TC treatment. It is reported that IGF2BP2 is overexpressed in selumetinib-resistant papillary TC cell lines and promotes ERBB2 translation efficacy by targeting ERBB2 mRNA, which contributes to the ERBB2-mediated acquired resistance to selumetinib [15]. At the same time, in radioiodine refractory papillary TC cells, IGF2BP2 is also highly expressed and is responsible for the insensitivity to radioiodine via the enhancement of RUNX2 mRNA stability an m6A-dependent manner, thus blocking the differentiation of these TC cells [85]. These findings imply that IGF2BP2 facilitates the resistance to chemotherapy and radiotherapy in TC, providing insights into overcoming therapeutic resistance in TC by combining drugs inhibiting IGF2BP2 with chemotherapy and radiotherapy. Besides, a most recent study reveals that m6A modification is associated with immune cell infiltration in the tumor microenvironment of TC, which can distinguish immuno-heat from immuno-cold phenotypes [86], suggesting that m6A dysregulation also influence immunotherapeutic responsiveness in TC.

It can be concluded that m6A dysregulation is related to drug resistance in TC. Targeting abnormal m6A regulators holds promising therapeutic significance by inhibiting tumor progression, attenuating chemoresistance and radioresistance, and potentiating immunotherapy in patients with TC. Currently, several small-molecule suppressors targeting m6A regulators have been developed for further clinical application. For example, in myeloid leukemia, small-molecule drugs STM2457 and FB23-2, inhibit the activity of METTL3 and FTO with high efficacy and selectivity [87, 88], and some of them have yielded encouraging preliminary findings in preclinical investigations. In this context, drugs and tactics that target m6A regulators have strong practical and clinical value in the treatment of TC. However, there are lacking of sufficient research data on the effects and mechanisms of m6A regulators in TC. Thus, identification of effective small-molecule compounds, traditional medicines or natural products targeting m6A regulators deserves further investigation in this disease, owing to their safety, hypotoxicity and accessibility.

## Conclusion

**Fig. 2:**
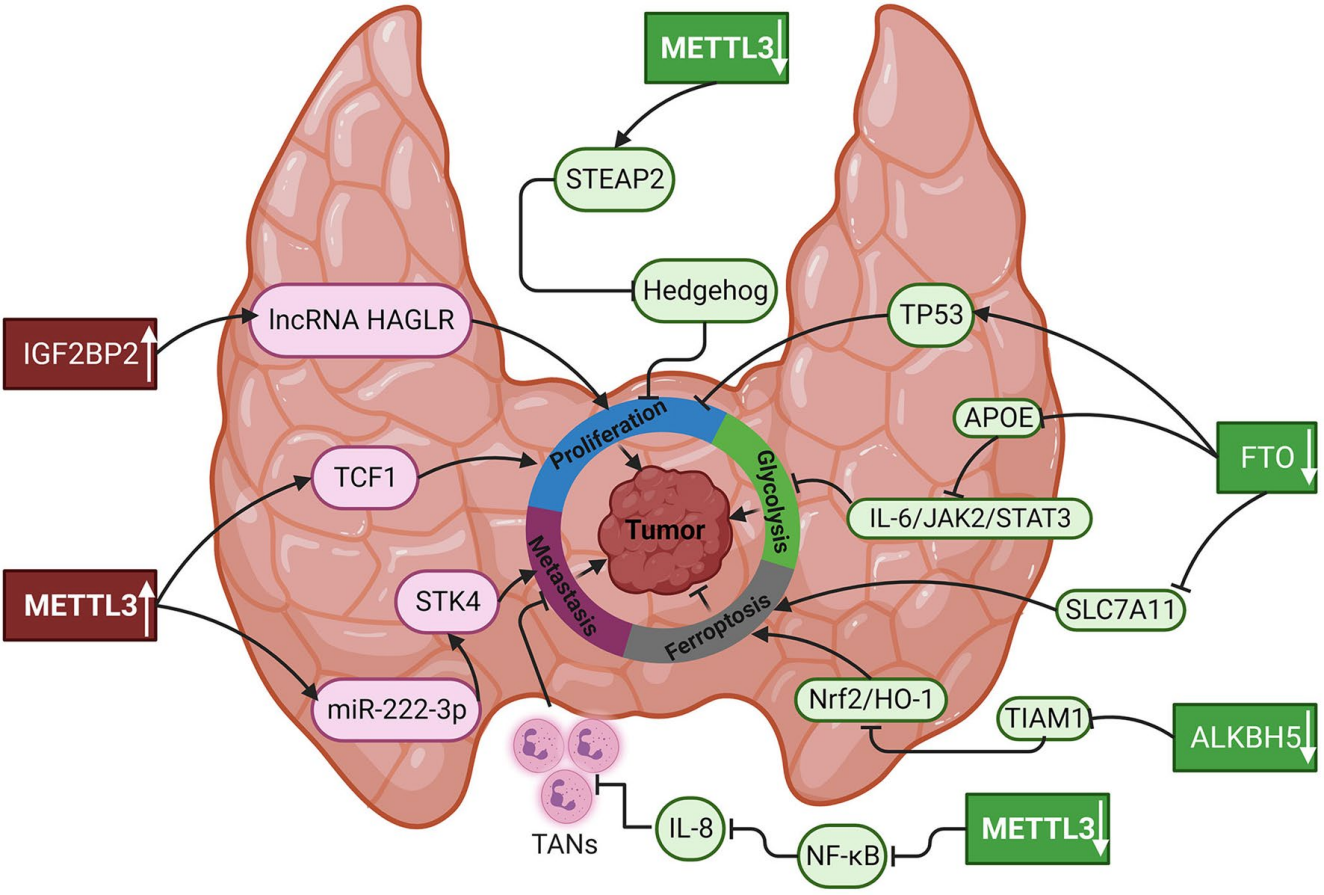
Role of the m6A modification in thyroid cancer. Oncogenic m6A regulators (red), METTL3 and IGF2BP2, are highly expressed in thyroid cancer, and promote cell proliferation and metastasis; while tumor-suppressive m6A regulators (green), including METTL3, FTO and ALKBH5, are downregulated in thyroid cancer, which inhibit tumor progression by suppressing proliferation, metastasis and glycolysis, as well as promoting ferroptosis. APOE, apolipoprotein E; STEAP2, six transmembrane epithelial antigen of the prostate 2; STK4, serine/threonine stress kinase 4; TANs, tumor-associated neutrophils; TCF1, T-cell factor 1; TIAM1, T-lymphoma invasion and metastasis. ↑indicates upregulation, ↓ indicates downregulation, → indicates a promoting effect and ⊥ indicates an inhibitory effect

This review stresses the importance of m6A modification in progression, prognosis and treatment of TC. This process is intricately modulated by a variety of regulators, including writers, erasers and reader proteins, which participate in oncogenesis and tumor development via affecting RNA metabolism and further altering oncogenes and tumor suppressors at the transcriptional and translational levels. Dysregulated expression of m6A regulators, including METTL3, FTO, ALKBH5 and IGF2BP2, has been implicated in TC proliferation, invasion, and metastasis by regulating biological processes, such as ferroptosis and glycolysis (Fig. 2). It should be noted that m6A-regulatory proteins either promote or suppress tumor progression, in a context-dependent manner. Thus, further investigations need to take TC models and histology subtypes into account. In addition, mediators that induce aberrant expression of m6A regulators in TC are still elusive. Clarification of these upstream regulators is crucial for understanding the pathogenesis and developing potential targets for the treatment of TC. The m6A modification is a dynamic and reversible process due to mutual influence on m6A regulators. It is essential to verify the molecular mechanism of m6A regulators interacting with each other during TC progression. Although various studies have demonstrated that dysregulated m6A regulators could be used as independent prognostic factors in TC, the optimal candidates for the early diagnosis and screening of TC are unknown. Identification of m6A regulator signatures through bioinformatics may provide diagnostic biomarkers and prognostic indicators, with high sensitivity and specificity, for patients with TC. Furthermore, targeting m6A regulators to inhibit tumor development and drug resistance may be promising for TC treatment. Some therapeutic strategies, such as small-molecule compounds, traditional medicines or natural products targeting m6A regulators, deserve to estimate their selectivity, safety and efficacy in the experimental setting for further clinical application.

## Data Availability

Not applicable.
